# Dyadic Associations between Daily Spousal Arguments and Affective Reactions: The Moderating Role of Emotion Work

**DOI:** 10.1093/geroni/igaf122.2932

**Published:** 2025-12-31

**Authors:** Dakota Witzel, Madeline Nichols, Kelly Chandler, Kelly Cichy, Robert Stawski

**Affiliations:** South Dakota State University, Brookings, South Dakota, United States; Oregon State University, Corvallis, Oregon, United States; Oregon State University, Corvallis, Oregon, United States; Kent State University, Stow, Ohio, United States; Utah State University, Logan, Utah, United States

## Abstract

Daily stressors, like arguments with spouses, are predictors for changes in same-day affect. Emotion work - defined as activities done to promote another’s positive emotional state - has been shown to have mixed associations with health and well-being. Notably, emotion work is more prevalent during stressful experiences. Using the 2015 wave of the Health and Relationships Study, we explored dyadic associations between daily arguments with spouses in gender-diverse marriages and both negative and positive affect and whether emotion work modified associations. Middle-aged couples (Ncouples = 378; 30% different-gender; Mage = 48.22, Agerange = 35-65) in same-gender and different-gender marriages completed 10 days of daily diary measurements on daily stressors, emotion work, and affect. Preliminary results using 3-level multilevel models suggest the odds of reporting an argument did not differ by gender composition (ps<.05), but did differ by the amount of emotion work by the respondent (OR = 3.88; p<.05), and their partner (OR = 2.51, p<.05). Significant differences in negative and positive reactivity emerged when respondents or their partners reported a spousal argument that day compared to non-argument days (ps<.05). On days when a spousal argument was reported, more emotion work from the respondent was related to greater negative and positive affective reactivity (ps<.05). Discussion will focus on the importance of spousal arguments and emotion work for shaping couple wellbeing, and their relevance throughout adulthood. Future work will aim to examine whether associations occur for longer-term affective reactions (e.g., affective residue) and differ by relationship composition (e.g., different-gender, same-gender).

